# Syndrome of Inappropriate Antidiuretic Hormone Secretion (SIADH) Complicated by Generalized Tonic-Clonic Seizures After a Course of Antibiotics: A Case Report

**DOI:** 10.7759/cureus.37607

**Published:** 2023-04-15

**Authors:** Mumtaz O Sanni, Jeyanthy Rajkanna, Satyanarayana V Sagi, Samson O Oyibo

**Affiliations:** 1 Medicine, Peterborough City Hospital, Peterborough, GBR; 2 Diabetes and Endocrinology, Peterborough City Hospital, Peterborough, GBR; 3 Endocrinology and Diabetes, Peterborough City Hospital, Peterborough, GBR

**Keywords:** hypertonic saline, hyponatremia, generalized tonic clonic seizures, siadh, cephalexin, nitrofurantoin

## Abstract

Nitrofurantoin and cephalexin are commonly used antibiotics for treating urinary tract infections. Hyponatremia secondary to syndrome of inappropriate antidiuretic hormone secretion (SIADH) has been reported as a rare side effect of nitrofurantoin but has never been a reported side effect of cephalexin. We report a case of a 48-year-old female who developed severe hyponatremia complicated by generalized tonic-clonic seizures after a course of antibiotics (nitrofurantoin followed by cephalexin) used for treating a urinary tract infection. She presented to the emergency department with a one-week history of dizziness, nausea, fatigue, and listlessness. She also had a two-week history of persistent urinary frequency despite completing a course of nitrofurantoin followed by a course of cephalexin. While in the emergency department waiting room, she had two episodes of generalized tonic-clonic seizures. Immediate post-ictal blood test results revealed severe hyponatremia and lactic acidosis. Results were consistent with severe SIADH and she was subsequently managed with hypertonic saline and fluid restriction. She was discharged after 48 hours of admission when her serum sodium levels normalized. Though we believe that nitrofurantoin was the culprit drug, we still asked the patient to avoid future use of both nitrofurantoin and cephalexin. Healthcare providers need to be aware of antibiotic-induced SIADH when assessing patients with hyponatremia.

## Introduction

Syndrome of inappropriate antidiuretic hormone secretion (SIADH) is characterized by inappropriate secretion of antidiuretic hormone in the presence of a normal plasma volume. This results in excess water and reduced serum sodium levels accompanied by inappropriately elevated urine osmolality [1]. The severity of SIADH depends on the speed of onset and the degree of hyponatremia - mild with serum sodium ranging from 130 to 135 mmol/L, moderate with serum sodium ranging from 125 to 129 mmol/L, and severe with serum sodium less than 125 mmol/L [1]. Symptoms of hyponatremia depend on the severity and include anorexia, nausea, listlessness, headaches, irritability, drowsiness, confusion, seizures, and coma due to cerebral edema [1]. Patients with mild hyponatremia are generally asymptomatic.

There are many causes of SIADH, the list includes medications, inflammatory neurological disorders, cancers, lung disease, surgery, and general anesthesia [2]. The Bartter-Schwartz criteria are used to make a diagnosis of SIADH, i.e., serum sodium level less than 135 mmol/L, serum osmolality less than 275 mOsm/kg, urinary sodium level more than 30 mmol/L, and urine osmolality >100 mOsm/L. There must be no volume depletion, no other causes of hyponatremia, no recent use of diuretic drugs, and the hyponatremia responds to fluid restriction [3]. The first-line therapy for SIADH is eliminating the cause and restricting fluid intake. For severe symptomatic hyponatremia, a weight-based calculated bolus of 3% hypertonic saline (sodium chloride) given intravenously with regular sodium monitoring is recommended [4].

Several medications have been implicated in medication-induced SIADH, but antibiotics are rarely on the list [5]. There are a handful of case reports implicating trimethoprim-sulfamethoxazole, ciproﬂoxacin, cefoperazone/sulbactam, rifabutin, azithromycin, and nitrofurantoin. However, these have occurred in elderly individuals, in the presence of other comorbidities, and concomitant use of diuretics and antidepressants [6].

We present a case of a female who developed severe SIADH complicated by generalized tonic-clonic seizures after completing a course of antibiotics (nitrofurantoin followed by cephalexin) for the treatment of a urinary tract infection.

## Case presentation

Medical history and demographics

A 48-year-old female presented to the emergency department with a one-week history of nausea, dizziness, fatigue, and listlessness, and a two-week history of urinary frequency. While waiting to be assessed, she had two self-limiting episodes of generalized, tonic-clonic seizures, the first lasting two minutes and the second lasting one minute. There was tongue biting and minor head trauma during the first seizure episode. She did not complain of fever, dysuria, or vomiting. In the 10 days prior to the presentation, she had completed a three-day course of nitrofurantoin, followed by a five-day course of cephalexin for the treatment of a urinary tract infection. However, her symptoms started after completing the course of nitrofurantoin. She had also increased her fluid intake by 50% during that period, thinking she could flush out the infection. She had no past medical history of epilepsy. She was not on any regular medication and had not taken any other medication apart from the above-mentioned antibiotics. She had never smoked, and she drank two units of alcohol per week.

On examination, she was alert but fatigued. She had a bruised tongue and a right-sided scalp wound. Her heart rate was 96 beats per minute, her respiratory rate was 16 breaths per minute, and she had an oxygen saturation of 96% on air. Her blood pressure was initially 193/84 mmHg during the post-ictal period, then settled at 118/75 mmHg. Her temperature was 37.2°C. Chest and abdominal examination revealed no abnormalities. There was no ankle edema. She weighed 52 kg.

Investigations

The initial investigations revealed severe hyponatremia with a low serum osmolality (Table 1). Urinalysis demonstrated concentrated urine, which was inappropriate in the presence of severe hyponatremia (Table 2). Results were suggestive of syndrome of inappropriate antidiuretic hormone secretion (SIADH). Arterial blood gas sampling demonstrated severe lactatemia and lactic acidosis during the post-ictal period (Table 3). Thyroid hormones, liver enzymes, and kidney function tests were normal. A chest x-ray demonstrated a region with an air-bronchogram in the left middle zone suggestive of aspiration pneumonitis, and her electrocardiography was normal. A computerized tomography scan of the head revealed a scalp hematoma only. A urine sample sent a day before admission yielded no bacterial growth.

**Table 1:** Initial blood test results.

Blood test	Result	Reference range
Sodium (mmol/L)	114	133-146
Potassium (mmol/L)	3.3	3.5-5.3
Chloride (mmol/L)	79	95-108
Creatinine (µmol/L)	46	59-104
Urea (mmol/L)	1.7	2.5-7.8
Bicarbonate (mmol/L)	17	22-29
Glucose (mmol/L)	12.2	4-7
Thyroid-stimulating hormone (mU/L)	1.1	0.3-4.2
Calcium (mmol/L)	2.16	2.2-2.6
Serum osmolality (mOsm/kg)	240	275-295
Albumin (g/L)	50	35-50
Early morning cortisol (nmol/L)	865	250-600
C-reactive protein (mg/L)	<1	<10
Hemoglobin (g/L)	128	130-180
White cell count (10^9^/L)	12.6	4.0-11.0
Platelet count (10^9^/L)	399	150-400

**Table 2:** Initial urine test results. SIADH: syndrome of inappropriate antidiuretic hormone secretion The blood and urine results meet the criteria for the diagnosis of SIADH.

Spot urine test	Result
Osmolality (mOsm/kg)	264
Sodium (mmol/L)	76
Potassium (mmol/L)	19
Urea (mmol/L)	41

**Table 3:** Arterial blood gas sampling. Results indicate severe lactic acidosis during the post-ictal period.

Arterial blood gas sampling	Result	Reference range
Arterial pH	7.216	7.35-7.45
Arterial PO_2_ (kPa)	10.09	10.67-13.3
Arterial PCO_2_ (kPa)	5.09	4.67-6.0
Arterial bicarbonate (mmol/L)	15.1	22-26
Base excess (mmol/L)	-11.9	-2-+2
Lactate (mmol/L)	14.22	0.20-1.80

Treatment

A bag of 0.9% sodium chloride was initially set up in the immediate post-ictal period and the patient was given an intravenous bolus of levetiracetam (2 g) to prevent further seizures. On getting the blood results indicating severe hyponatremia, the infusion was changed to a bolus of 5% hypertonic saline at a dose of 100 mL over one hour to increase the serum sodium level by 4-5 mmol/L, thus preventing cerebral edema and further seizures. Her acid-base balance returned to normal within a few hours. The serum sodium level went up to 119 mmol/L after the bolus infusion of hypertonic saline. A slow 0.9% saline infusion was put up to keep the line open. The patient was advised to restrict fluid intake to less than 1.5 liters a day. Her serum sodium was 123 mmol/L the next day and 134 mmol/L the day after. She was discharged from the hospital the next day.

Outcome and follow-up

The patient continued to do well and her follow-up serum electrolytes were normal. This case was reported to the Medicines and Healthcare Products Regulatory Agency (MHRA), United Kingdom. Even though we believed that nitrofurantoin was the culprit drug, we still asked the patient to avoid future use of nitrofurantoin and cephalexin.

## Discussion

We have presented a case of a female who developed severe SIADH complicated by generalized tonic-clonic seizures after completing a course of antibiotics (nitrofurantoin followed by cephalexin) for the treatment of a urinary tract infection. She had no signs of infection, and she had not ingested any other medication that would cause SIADH or hyponatremia. Her symptoms started after completing the course of nitrofurantoin. She was found to have severe hypoosmolar hyponatremia. She had also increased her fluid intake by 50%, which most likely exacerbated the hyponatremia. Her biochemistry improved rapidly after a calculated dose of intravenous hypertonic saline and fluid restriction.

In patients with severe hyponatremia and severe symptoms, it is recommended that they receive 150 mL of 3% hypertonic saline over 20 minutes in the first hour of management, regardless of whether the hyponatremia is acute or chronic [4]. Then repeat blood test after 20 minutes, and if serum sodium levels have not increased by 5 mmol/L, a repeat bolus of 150 mL 3% hypertonic saline over 20 minutes is given. A follow-up with a very slow infusion of 0.9% saline, to keep the line open is recommended once serum sodium levels have risen by 5 mmol/L [4]. Guidelines also recommend that the rise in serum sodium level should not exceed a total of 10 mmol/L in the first 24 hours, and should not exceed 8 mmol/L per day during subsequent days while the serum sodium is still less than 130 mmol/L.

We did not have 3% hypertonic saline available, so our patient received a bolus of 5% hypertonic saline at a dose of 100 mL over one hour, and a repeat blood test was performed thereafter. A slow infusion of 0.9% saline was put up to keep the line open. Our patient did not require another bolus infusion of hypertonic saline (100 mL of 5% hypertonic saline contains 85.6 mmol of sodium compared to 77 mmol contained in 150 mL of 3% hypertonic saline).

Nitrofurantoin is a synthetic antimicrobial widely used to treat lower urinary tract infections [7]. Since the emergence of increasing resistance to newer antibiotics, such as trimethoprim-sulfamethoxazole and beta-lactam antibiotics, several major guidelines have recommended nitrofurantoin as first-line therapy for treating uncomplicated lower urinary tract infections [7]. The most commonly reported side effects of nitrofurantoin are nausea, vomiting, loss of appetite, and diarrhea. Rare side effects are pulmonary toxicity (pneumonitis), liver injury, and peripheral neuropathy. There is no mention of hyponatremia or SIADH being a recognized side effect of nitrofurantoin in the product literature. The use of nitrofurantoin is contraindicated in women in the later part of pregnancy and in labor, in neonates younger than one month of age, and in patients with significant renal impairment.

A literature search revealed only one previous case report of an 87-year-old female who developed generalized tonic-clonic seizures three days after commencing nitrofurantoin for a urinary tract infection. She was found to have severe hyponatremia and biochemical features of SIADH. This patient also had a history of Alzheimer’s disease and hypertension and was taking metoprolol, ramipril, and furosemide [8]. Therefore, our case report is the second to report nitrofurantoin-induced hyponatremia complicated by tonic-clonic seizures. Our patient was much younger, had no comorbidities, and was not on any medication that can contribute to hyponatremia.

Our patient also had a course of cephalexin before attending the emergency department. Cephalexin is a first-generation cephalosporin used for treating urinary tract infections. There is no mention of hyponatremia being a side effect, and there are no case reports implicating cephalexin as a cause of hyponatremia. There is only one case report of a 70-year-old woman who developed hyponatremia while on an intravenous course of cefoperazone/sulbactam on two separate occasions [9]. Cefoperazone is a third-generation cephalosporin, which is combined with a beta-lactamase inhibitor, sulbactam. As previously mentioned, our patient’s symptoms of hyponatremia started before commencing cephalexin.

## Conclusions

There are rare reports of antibiotics causing SIADH with severe hyponatremia complicated by tonic-clonic seizures. Though the patient in this case report presented after the completion of nitrofurantoin and cephalexin, her hyponatremic symptoms started after the completion of nitrofurantoin; before starting the cephalexin. We, therefore, believe that nitrofurantoin was the cause of severe hyponatremia in this case. It may be prudent to consider asking patients on nitrofurantoin to look out for symptoms of hyponatremia. Healthcare providers need to be aware of antibiotic-induced SIADH when assessing patients with hyponatremia.
